# Studies on Labour Safety in Construction Sites

**DOI:** 10.1155/2015/590810

**Published:** 2015-12-29

**Authors:** S. Kanchana, P. Sivaprakash, Sebastian Joseph

**Affiliations:** ^1^Department of Civil Engineering, RVS Technical Campus, Coimbatore 641402, India; ^2^Department of Mechanical Engineering, A.S.L. Pauls College of Engineering and Technology, Coimbatore 641032, India; ^3^Department of Mechanical Engineering, Karpagam University, Coimbatore 641021, India

## Abstract

Construction industry has accomplished extensive growth worldwide particularly in past few decades. For a construction project to be successful, safety of the structures as well as that of the personnel is of utmost importance. The safety issues are to be considered right from the design stage till the completion and handing over of the structure. Construction industry employs skilled and unskilled labourers subject to construction site accidents and health risks. A proper coordination between contractors, clients, and workforce is needed for safe work conditions which are very much lacking in Indian construction companies. Though labour safety laws are available, the numerous accidents taking place at construction sites are continuing. Management commitment towards health and safety of the workers is also lagging. A detailed literature study was carried out to understand the causes of accidents, preventive measures, and development of safe work environment. This paper presents the results of a questionnaire survey, which was distributed among various categories of construction workers in Kerala region. The paper examines and discusses in detail the total working hours, work shifts, nativity of the workers, number of accidents, and type of injuries taking place in small and large construction sites.

## 1. Introduction

In India, construction industry is the second largest employer when compared to agriculture [1]. Throughout the world, the construction area of civil engineering is one of the most hazardous industries. The number of fatal accidents taking place at the construction sites is quite alarming and the major cause was found to be fall of persons from height and through openings [2].

In the present scenario, the Indian construction industry is quite large and complex involving latest technology as well as man power. On a par with the development of construction industry, drawbacks in terms of safety and health aspects are also witnessed.

The Indian construction labour force is 7.5% of the total world labour force and it contributes to 16.4% of fatal global occupational accidents [3]. In the construction industry the possibility of a fatality is five times more likely than in a manufacturing industry, whereas the risk of a major injury is two and a half times higher. India has the world's highest accident rate among construction workers, according to a recent study by the International Labour Organization (ILO) that cited one survey by a local aid group showing that 165 out of every 1,000 workers are injured on the job [4]. Construction workers are not the only sufferers of accidents but also the public including children are affected. These accidents diminish the image of the construction industry, and as a result there is shortage of skilled labour [5].

In the past few decades, need for safety awareness among construction industries was realized [6]. This is due to the high cost associated with work related injuries, workers compensation, insurance premium, indirect costs of injuries, and litigation. Every year, a considerable amount of time is lost due to work related health issues and site accidents [7]. There are several factors responsible for health problems and construction site accidents. From the result of Occupational Safety and Health Administration examination on the causes of construction fatalities, it was shown that 39.9% of fatalities in construction were caused by falls, 8.4% were struck by objects, 1.4% were caught in between incidents, and 8.5% were electrocution [8]. There are several techniques that can be adopted for labour safety such as safety organization and management, safety policy, safety organization, safety training, safety committees, site layout, first aid, lighting, personal protective equipment, and welfare facilities.

Lack of communication among the various departments involved and lack of proper inspections are the major reasons for accidents occurring at a construction sites. The construction site accidents may be caused due to the factors such as collapse of building parts and masses of earth, falling of objects and pieces of work on workers, fall of persons from heights, ladders, and stairs, loading, unloading, and transportation of loads, working on machines, and blasting with explosives.

Every effort must be taken to bring up the level of consciousness among the employees as well as management about the importance of health and safety at work sites [9]. It is highly desirable to decrease the rate of labour accidents for employee working in the construction industry all over the world. Many preventive measures to address this problem have been proposed and carried out. However, accidents keep occurring with depressing regularity. Hence, new effective measures for prevention of labour accidents are always keenly anticipated.

Construction projects carried out in large scale are following good safety measures as a separate safety department is available in these companies. But small scale projects taken up by local contractors are not aware of the safety requirements that could prevent construction site accidents. Preventing labour accidents, occupational illness, and injuries should be a primary concern of all employers. The paper examines the current status of safety at workplace and to create a safe working environment for the employees of construction companies. The study included physical visit to different construction sites, collecting the data and feedback regarding number of workers, nativity of the workers, total work hours, and work shifts from construction site workers using questionnaire. Information pertaining to the number of accidents taking place in small and large construction sites, cause for the accidents, and type of injuries suffered by the workers was collected and examined.

## 2. Literature Review

The construction industry is a very dangerous industry. The performance of the industry in occupational health and safety is very poor. The standard of occupational health and safety is even worse in developing countries. In Indian construction industry OHS has never been given prime importance. Even though in India construction industry is significantly booming, there are no proper initiatives undertaken by the government to implement OHS rules and regulations.

Huang and Hinze analyzed accident caused due to fall of workers at construction sites and the result showed that most fall accidents took place at elevations of less than 9.15 m, occurring primarily on new construction projects of commercial buildings and residential projects of relatively low construction cost [10].

Jannadi and Bu-Khamsin conducted questionnaire survey among industrial contractors in the Eastern Province of Saudi Arabia and formal interviews with the contractors and officials responsible for construction safety were taken. 72% of the companies participated in this survey were the general building construction companies [11]. Twenty main factors and eighty-five subfactors and their level of importance based on the survey results and analysis were identified. Pheng and Shiua emphasized that integration between quality and safety should be achieved for better coordination and utilization of resources [12]. Koehn and Datta through their study concluded that safety rules and regulations not only overcome issues like poor quality work, unsafe working conditions, and lack of environmental control but also reduce cost and enhance productivity [13]. Wilson Jr. and Koehn suggested that safety practices vary with construction sites, as every site has unique safety aspects. Larger construction projects are better organized whereas small to medium firms do not have an adequate safety program or person to oversee safety criteria [6].

In developed countries, recent advancement in technology, on one hand, has contributed positively to industry productivity, but, on the other hand, it has created a more challenging and unsafe work environment [14]. Every construction worker is likely to be temporarily unfit for work at some time as a result of a minor injury or a health problem after working on a construction site [15]. Between 1989 and 1992, 256 people were fatally injured in the Australian Construction Industry. Statistics revealed that the fatality rate was 10.4 per 100,000 workers, which was similar to the fatality rate for road accidents [16]. It is estimated that, in China, every year, 3,000 workers belonging to construction sector were killed in work related accidents [17]. From a study conducted by Egyptian construction industry, it was concluded that safety programs organized by Egypt contractors were less formal and the accident insurance costs were fixed irrespective of the contractor's safety performance [18]. The most common cause of injuries and death in the construction sector is falling from heights [19]. The main causes include working on a scaffold or platform without guard rails, or without a safety harness correctly attached, and fragile roofs and ladders that are badly maintained, positioned, and secured. Slips, trips, and falls are the largest cause of accidents in all sectors [20].

In India, departments under the Ministry of Labour and Employment deal with OSH issues in construction sector under the head of Chief Labour Commissioner. Directorate General Factory Advise Service Labour Institute (DGFASLI) provides technical support in drafting model rules, carrying out surveys, and conducting training programmes in construction sector. A number of Labour Laws are applicable to the workers engaged at construction sites. These are as follows:

(i) Contract Labour (Regulation & Operative) Act, 1970,
(ii) Minimum Wages Act, 1948,
(iii) Payment of Wages Act, 1936,
(iv) Equal Remuneration Act, 1976,
(v) Inter-State Migrant Workmen (Regulation of Employment and Condition of Services) Act, 1979,
(vi) The Building and Other Construction Workers Act, 1996.

The Building and Other Construction Workers (Regulation of Employment and Conditions of Service) Act, 1996, was enacted on 1.3.1996. The act is applicable to all establishments employing 10 or more workers in any building and other construction works. The Chief Labour Commissioner is entrusted with the task of enforcement of this act and the central rules [21].

## 3. Methodology

The methodology is designed in order to reflect the different aspects of construction sites and to reflect overall project objectives.

As the first step, a detailed questionnaire is designed in order to quantify the criteria influencing the safety at site with weight-age depending upon its importance. The criteria considered for survey are as follows:

(i) labour information: position, number of workers, work shift, and timing,
(ii) accident evaluation: number of accidents, type of injury, and reason for accidents.

In the next two steps, the questionnaire is distributed and filled questionnaire is collected back from respondents. A total of 127 interviews were conducted across 52 sites with a range of managers, site engineers, personnel responsible for safety, and labourers.  Table 1 shows details of number of interviews.

In the last step, findings based on the questionnaire and interviews were used to analyze the safety performance of the construction industry.

## 4. Results and Discussion

### 4.1. Labour Information, Number of Floors, Work Shift, and Timing

The data collection was done from 32 residential building sites, 16 commercial building sites, 2 educational building sites, and 2 religious buildings sites (residential buildings like houses, hostels, and apartments; commercial buildings like shops, office, bank, and auditorium; educational buildings like school, college, and tuition centers).

In small industries only G+0 and G+1 building construction were found on sites. Data were collected from 11 numbers of G+0 building sites and 16 numbers of G+1 building sites. In large industries data collection was done from 12 residential buildings, 10 commercial buildings, and 9 educational buildings. 10 numbers of ≤G+2 building, 9 numbers of G+3 ≤ G+6 building, and 6 numbers of ≥G+7 building details were collected. Only day works were found on small construction sites. Maximum numbers of works start at 8 AM and finish at 6 PM. The working time is comfortable in all sites with two hours of interval time for food and rest. In large industries more than G+2 constructions are found in large number. In some sites night shift works are done to complete the project on time.

### 4.2. Number of Workers

It was found that the average age of the workers was 32 years, with 37% of sample being younger than 39 years.  Table 2 shows the total number of workers in small and large construction sites.

In small industrial sites 96 migrant workers and 337 Kerala workers are included. In large industrial sites 196 migrant workers and 489 Kerala workers are included. More numbers of migrant labourers are from Bengal. The migrant labourers are ready to work for low wages so that the contractors are happy to give job to them.

### 4.3. Accident Evaluation

#### 4.3.1. Number of Accidents, Type of Injury, and Reason for Accidents

Details of labour accident which happened in previous year were collected and rate of accident in each site is calculated.  Table 3 shows the details.

#### 4.3.2. Small Construction Sites

The total number of accidents happened in 27 small construction sites; the number of accidents in each category and its reasons are shown in Table 4. The average number of accidents was found to be 16.03 and the rate of occurrence of accident with respect to total number of persons was 62.8%.

**Figure 1:**
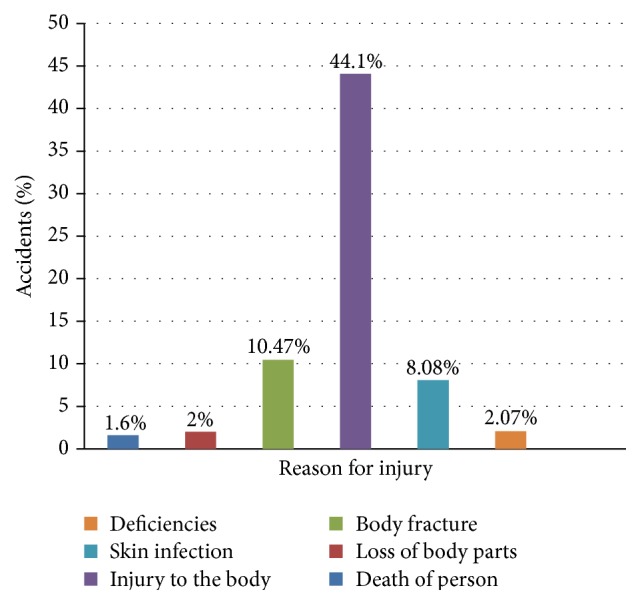
Percentage of accidents in small construction sites.

Figure 1 shows the percentage of accident in small construction sites in each category; the death of persons is 1.60%; loss of body parts is 2%; body fracture is 10.47%; injury to the body is 44.10%; skin infection is 8.08%; deficiencies to ear and eye are 2.07%. The average number of accidents is 10.07.

**Figure 2:**
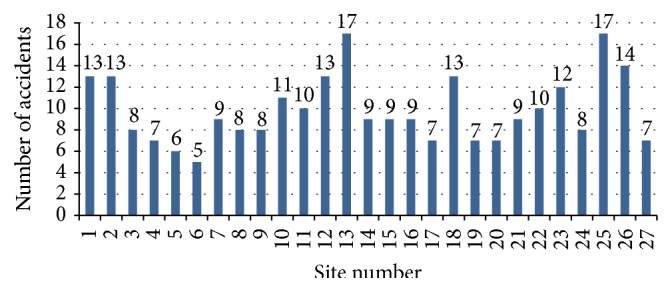
Number of accidents in small construction sites.

Figure 2 shows the total number of accidents in each small construction site. Site number 13 and site number 25 show more number of accidents as 17 and site number 6 shows less number of accidents as 5.

#### 4.3.3. Large Construction Sites

The total number of 325 accidents happened in 25 sites; the number of accidents in each category and its reasons are shown in Table 5.

**Figure 3:**
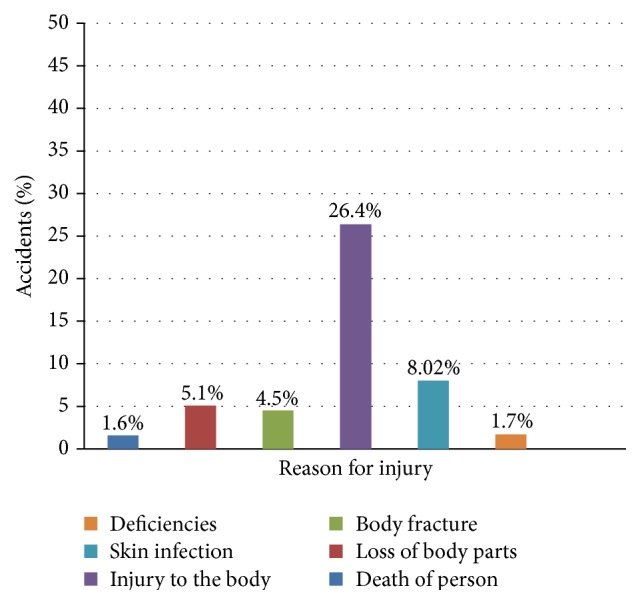
Percentage of accidents in large construction sites.

Figure 3 shows the percentage of accident in large construction sites in each category; the death of persons is 1.60%; loss of body parts is 5.1%; body fracture is 4.5%; injury to the body is 26.4%; skin infection is 8.02%; deficiencies to ear and eye are 1.7%. The average number of accidents is 13.

**Figure 4:**
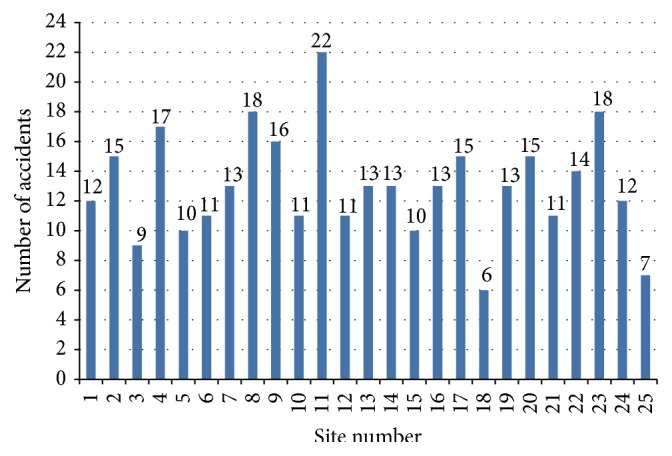
Number of accidents in large construction sites.

Figure 4 shows the total number of accidents in each large construction site. Site number 11 shows more number of accidents as 22 and site number 18 shows less number of accidents as 6.

A comparative study between labour safety in small construction industry and large construction industry is done and the details are shown in Table 6.

## 5. Conclusion

Owing to increase in complexity of operations, the construction industry has become more dangerous. Construction industries are faced with the challenge of having close monitor of their labour safety management systems to minimize occupational hazards. This paper is concluded with few points given below.

(i) The working time is comfortable for all categories of workers in most of the sites.
(ii) The average age of the workers was found be 32 years.
(iii) Maximum numbers of workers were native workers but migrant workers are ready to work for low wages.
(iv) The average number of accidents was found to be 16.03 in small construction sites.
(v) The average number of accidents was found to be 13.00 in large construction sites.
(vi) In both small and large construction sites, more number of accidents occurred due to body injuries accounting to 44.1% and 26.4%, respectively.
(vii) In general, safety of workers in all construction is to be improved.
(viii) Contractors and owners must give utmost importance to the safety of the workers.

The paper concluded that the major cause for construction accidents is due to injuries. Further studies can be conducted on such injuries and methods that can be adopted to prevent such injuries. Employer can always check legislation and draw up a proper health and safety plan specific to employer's workplace and employees. The provisions available in the laws that can be followed by employers for ensuring safe construction site environment can be studied in detail.

## Figures and Tables

**Table 1:** Interview details.

Position of person	Number of persons interviewed
Site Manager	5
Site Engineer	20
Safety Officer	4
Contractors	8
Labourers	90

**Table 2:** Total number of workers in small and large construction sites.

Site number	Small construction sites		Large construction sites	
	Keralites	Non-Keralites	Keralites	Non-Keralites
1	12	3	18	5
2	12	3	15	4
3	15	5	10	7
4	15	5	25	14
5	12	6	28	15
6	12	6	21	7
7	15	9	30	6
8	15	9	26	18
9	14	2	25	20
10	14	2	20	15
11	12	8	16	4
12	12	8	20	5
13	14	4	18	15
14	14	4	14	13
15	16	0	20	9
16	16	0	16	5
17	15	2	15	4
18	15	2	17	3
19	11	3	20	4
20	11	3	19	4
21	14	3	20	9
22	14	3	18	5
23	16	0	20	6
24	16	0	22	6
25	12	3	16	3
26	12	3	0	0
27	13	4	0	0

**Table 3:** Key words to represent accident.

TI = type of injury	RI = reason for injury
a = lack of training b = lack of care of labour (injured person) c = lack of care of another labour d = unsafe scaffolding, ladder, machines, vehicles e = did not use PPE, f = environmental factor g = lack of site inspection, h = safety symbols are not used i = lack of safety arrangements j = unsafe site planning and layout k = improper labour facilities, l = electrical shock m = failures of structure, n = due to use of alcohol o = other reasons, *N* = number of accidents	I = death of person II = loss of body parts III = body fracture IV = injury to the body V = skin infection VI = deficiencies to ear and eye VII = any other

**Table 4:** Overall accident details in small construction sites.

RI	TI															
	a	b	c	d	e	f	g	h	i	j	k	l	m	n	o	*N*
I	√	√	√	√	√		√	√	√	√		√	√	√		7
II	√		√	√									√			10
III	√	√		√		√	√		√					√		20
IV	√	√		√	√		√		√					√		91
V					√				√		√					35
VI		√		√										√		9
Total																= 172

**Table 5:** Overall accident details in large construction sites.

RI	TI															
	a	b	c	d	e	f	g	h	i	j	k	l	m	n	o	*N*
Death of person	√	√	√	√	√		√	√	√	√		√	√	√		11
Loss of body parts	√		√	√									√			35
Body fracture	√	√		√		√	√		√					√		31
Injury to the body	√	√		√	√		√		√					√		181
Skin infection					√				√		√					55
Deficiencies to ear and eye		√		√										√		12
Total																= 325

**Table 6:** Comparative study on small and large construction sites.

Site number	Study criteria	Small construction sites	Large construction sites
1	Number of construction sites	27	25

2	Number of questionnaires collected	48	79

3	Average number of accidents in one site per year	16.03	13

4	Rate of occurrence of accidents with respect to total number of persons	62.8%	47.44%
